# Multivariate Comparison of Cytokine Profiles for Normal- and Low-Bone-Density Subjects

**DOI:** 10.3390/diagnostics9040134

**Published:** 2019-09-30

**Authors:** Kamaludin Dingle, Fawaz Azizieh

**Affiliations:** Centre for Applied Mathematics and Bioinformatics, Department of Mathematics and Natural Sciences, Gulf University for Science and Technology, P.O. Box 7207, Hawally 32093, Kuwait

**Keywords:** cytokines, osteoporosis, biomarkers, multivariate data analysis

## Abstract

Osteoporosis is a serious worldwide public health concern. The role of the immune system in the onset of osteoporosis in postmenopausal women is an area of current research. Here we study data from a panel of 10 cytokines obtained from postmenopausal women, with both normal and low bone mineral density (BMD). Normal- and low-BMD groups are compared and contrasted, and further low-BMD participants are sub-classified into osteopenic and osteoporotic based on BMD levels, and compared to each other. Via the use of multivariate statistical tools, we examine contrasting groups in relation to: (a) the presence of subgroups/clusters; (b) whether groups have statistically different multivariate distributions; (c) how strongly groups differ (if at all), which relates to the practical/clinical significant of any differences; and (d) which cytokines contribute most to any differences between groups. We find that the normal- vs. low-BMD groups are markedly different (*p*-value = 0.00013), with IL-23, IL-12, TNF-*α*, IL-4 and IL-6 being the most important differentiating cytokines. No significant difference between the osteopenic and osteoporotic groups is found (*p*-value = 0.81). These findings may aid the development of cytokine therapies for osteoporosis, and suggest the use of certain cytokine profiles as biomarkers for osteoporosis risk factors, and ways to quantify the progress of treatment therapies.

## 1. Introduction

Osteoporosis is defined as a skeletal disorder characterized by compromised bone strength predisposing a person to an increased risk of fracture. With increasing life expectancy, postmenopausal osteoporosis is becoming a major worldwide health problem. According to statistics from the International Osteoporosis Foundation, worldwide, 200 million women are affected by osteoporosis [1]. Therefore, postmenopausal osteoporosis continues to pose a significant challenge.

Although estrogen is established to have direct effects on bone cells, what remains under investigation are other factors that may exacerbate bone loss at and after menopause, contributing to osteoporosis. The term “osteoimmunology” was first used in 2000 by Aaron and Choi to highlight the close and intricate communication between the immune and skeletal systems especially as observed in autoimmune and other inflammatory diseases [2]. Major advances and discoveries in this interdisciplinary research field have led to the recognition of molecular mechanisms, as well as various cytokines and other molecular signals, participating in the regulatory crosstalk between immune and bone cells.

Recent clinical and molecular evidence suggests that inflammation also exerts significant influence on bone turnover, inducing osteoporosis. Numerous proinflammatory cytokines have been implicated in the regulation of osteoblasts and osteoclasts, and a shift towards an activated immune profile has been hypothesized as an important risk factor [3]. Chronic inflammation and the immune system remodeling characteristic of aging, as well as of other pathological conditions commonly associated with osteoporosis, may also be determinant pathogenetic factors. In this context, it should be mentioned that the aging process is characterized by a progressive proinflammatory status, a phenomenon referred to as “inflamm-aging” by Franceschi et al. [4]. Furthermore, osteoclast-mediated bone loss has been reported in various inflammatory and autoimmune diseases such as rheumatoid arthritis, diabetes mellitus, lupus erythematosus, periodontal diseases and chronic viral infections such as human immunodeficiency virus [5,6,7,8,9,10].

Several cytokines are known to regulate the functions of osteoblasts, osteoclast progenitor cells and mature osteoclasts [11,12]. Inflammatory pro-resorptive cytokines that stimulate osteoclastogenesis and promote bone resorption include tumor necrosis factor-alpha TNF-*α*, interleukin IL-1, IL-8, IL-6, IL-12, IL-17 and IL-20, while anti-resorptive cytokines are tumor growth factor-beta TGF-*β*, interferon-gamma IFN-*γ* IL-4, IL-10, IL-13 and IL-23 [3,11,12]. However, some of these cytokines may possess dual pro-resorptive and anti-resorptive properties depending on the specific pathophysiological condition of the bone in vivo or the developmental stage of the osteoclast in vitro (reviewed in [11]). By extension it has been suggested that a shift in overall immune balance towards an activated inflammatory immune status may be an important risk factor for osteoporosis [3,13,14].

However, there is a complex network linking the different cytokines involved in immune mediated bone remodeling. Lymphocyte activation does not always lead to osteoporosis, because the final result depends on the specific cytokines produced and their reciprocal interactions. For example, IL-4 is one of the cytokines that inhibit osteoporosis. Moreover, its hyperproduction characterizes an atopic background and stimulates IgE synthesis [15]. Bone mineral density (BMD) in allergic patients who have not undergone cortisone therapy resulted in higher BMD compared to sex and age-matched healthy controls. On the other hand, antigen presenting cells (APC), in addition to stimulating bone resorption, could negatively regulate osteoclastogenesis through up-regulation of the RANK-L decoy receptor OPG.

Considering these complex interrelationships and the high potential for developing innovative new therapeutic drugs targeting osteoclastogenesis-regulating cytokines, there is a great need for better understanding cytokine profiles rather than individual ones. Our research aims to shed more light on this important but still poorly understood nexus between the immune system and skeletal system, with particular focus on osteoporosis. Additionally, while others have studied the links between cytokine concentration levels and osteoporosis, such studies often compare individual levels or ratios of a small number of cytokines. Given that cytokines form a network of interacting entities and that a single cytokine or a ratio of two may not provide sufficient information about the overall immune reactivity, it is of interest to study the combined levels of several cytokines as this may provide a better picture of immune reactivity. Indeed, we and others have advocated multivariate and advanced statistical methods in cytokine data analysis studies [16,17,18].

Furthermore, often cytokine studies focus on whether statistically significant differences between groups of normal vs. complication groups could be found, by for example, comparing the median cytokine concentration values of each group. However, merely statistical significance via finding a small *p*-value is not the whole story [17,19]; it is also very interesting to know if the values strongly differ, or are only slightly different, despite having small *p*-values. Indeed, we should consider the practical significance, not just the statistical significance, of the difference in cytokine values between groups. Practical, or clinical, significance can be expected to be related to the actual size of the difference; if the difference is small (but statistically significant), this suggests little practical or clinical significance or benefit. On the other hand, if the difference is large, then it suggests practical significance, such as the ability of a drug to alleviate symptoms. Furthermore, multivariate cytokine profile analysis may also suggest cytokine *importances* [17] which are a mathematical measure of the contribution of individual cytokines in separating one group from its comparable/matching control group.

In consideration of the above points, here we report a comparison of profiles of cytokines produced by mitogen-stimulated peripheral blood mononuclear cells from postmenopausal women with normal BMD versus osteopenic and osteoporotic postmenopausal women. We study previously published [20] data for 10 cytokines (TNF-*α*, IFN-*γ*, IL-10, IL-6, IL-17, IL-13, IL-12, IL-20, IL-23 and IL-4) from 71 postmenopausal women divided into four groups: normal (N) with *n* = 25 samples, low bone mass density (L) with *n* = 46, with the latter split into two further groups, namely osteopenic (OSN) with *n* = 31, and osteoporotic (OSR) with *n* = 15. Specifically, our analysis and this article is structured as follows: First, we analyze these data looking for the presence of multiple subgroups/clusters; second, whether there is statistical evidence for differences in the multivariate distributions of contrasting groups; third, we study the extent, or strength, of any differences between groups; fourthly, we compute cytokine importances. Finally, we discuss our results, suggests some conclusions and elaborate on limitations.

## 2. Results

### 2.1. Searching for Subgroups (Clusters)

We begin our analysis by looking for evidence of subgroups, or clusters, in each dataset. It is important to begin with this analysis because the presence of separated data clusters within a single medical group would alter the way subsequent statistical analysis is performed (e.g., treating separate clusters individually). Additionally, multiple clusters may have implications for a basic understanding of the disease, such as multiple different causes for a disease.

Here we investigate each of the N, L, OSN and OSR groups separately, counting the number of subgroups. Using the Gap Statistic [21] (Methods) we did not find evidence of separated subgroups/clusters in any of the groups. Having said that, cluster analysis with such small sample sizes is difficult, and so it may be that clusters could be found in larger samples.

### 2.2. Are the Groups Different?

Next we quantify whether contrasting groups have a statistically significant differences in their multivariate distributions. We compare N to L, and then OSN to OSR. We use the multivariate non-parametric Cramer test [22] (Methods), which is based on comparing the average between-group distance of samples to the average intra-group distances. In theory, this test should be able to detect a statistically significant difference if at least one variable is different between the groups. Because this test returns a single *p*-value on comparing two multivariate data sets, another advantage is that it circumvents the problem of having to adjust *p*-values when performing multiple comparisons. Indeed, when Azizieh et al. [20] studied this same N and L data set, nearly all the 10 individual cytokines were found to be different between the groups, with significant *p*-values of < 0.05. However, when reevaluating these *p*-values and using the Bonferroni correction [23] to adjust for the multiple comparisons, only *one* of the cytokines is found to be statistically significantly different between the groups. This discrepancy leaves a question mark regarding the difference, if any, between the N and L groups. Finally, the Cramer test can detect more complex differences between the distributions, such as correlations, which may not be easily detectable via univariate analysis, or even studying by ratios. Hence, while Azizieh et al. [20] did not find differences between the OSN and OSR groups, it is possible that the Cramer test may do.

On comparing the N and L samples, we find a highly statistically significant difference, with *p*-value = 0.00013 ≪0.05. Hence we can conclude that the N and L groups are indeed different, with strong statistical evidence. As for comparing OSN to OSR, the Cramer test found no statistically significant difference between the groups (*p*-value = 0.81 > 0.05), agreeing with the finding of Azizieh et al.

### 2.3. How Strongly Do the Groups Differ?

Now we investigate how strongly groups differ, which relates to the clinical significance of any differences found. To investigate how strongly groups differ, we will employ two methods, data projection and classification performance. We make PLS-DA projection plots (Methods) of N and L, and then OSN and OSR. These plots allow us to visualize the 10-cytokine data via a ‘shadow’ of the data on only two axes (dimensions), which are called *latent variable* 1 (LV1) and *latent variable* 2 (LV2). While there are many ways to project 10 variables onto only 2, this PLS-DA algorithm projects the data onto axes chosen in a way to maximize the separation between the groups.

**Figure 1:**
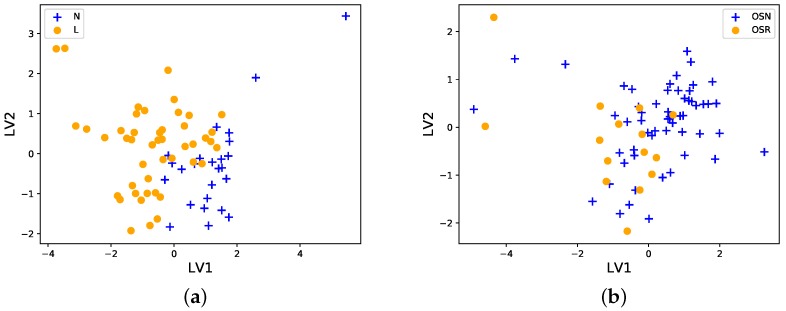
PLS-DA projections of the data onto two axes, latent variable 1 (LV1) and latent variable 2 (LV2). (**a**) The N (blue crosses) and L (yellow circles) groups do not overlap strongly, and hence are clearly different, but at the same time they are very close to each other with some overlap. (**b**) The OSN (blue crosses) and OSR (yellow circles) groups can be seen to be strongly overlapping, and hence not significantly different from each other. The data were log10 transformed, and centered and scaled before projection.

As can be seen in Figure 1a, the N and L groups are quite well separated, but still in ‘close’ proximity to each other, and with some overlap between the data samples. This suggests that the statistically significant difference found by the Cramer test above is large enough to be practically significant as well. Turning to the OSN and OSR samples, Figure 1b shows that the groups overlap strongly, as we might predict from the non-significant *p*-value obtained by the Cramer test.

While visual examination of data is very important, viewing multivariate data projections may also be misleading, due to the loss of information when projecting the data. Also, it is a qualitative rather than quantitative approach to comparing groups. Therefore, we next examine how strongly contrasting groups differ from another perspective, which is the ability of a classification algorithm to correctly predict group samples, a method we used earlier [17]. Intuitively, if a statistical classification algorithm can easily predict which group a given sample belongs to, then the groups must have markedly different cytokine profiles. We will use the partial least square regression (PLSR) classification algorithm here, and use leave-one-out cross validation to predict the class of each sample in the groups. We use the standard receiver operator characteristic area under the curve (ROC AUC) to measure classification ability. The ROC AUC metric ranges between 0 and 1, with scores ≈ 0.5 indicating very poor classification ability (no better than random guessing), and ≈1 indicating near perfect classification ability.

On comparing N and L groups, we find an ROC AUC value of 0.90 which is a high score (with 95% confidence interval from bootstrap sampling [0.82,0.96]), thereby quantitatively implying that the N and L groups are quite clearly separate. Again, this suggests a practical and clinically relevant difference between the N and L groups. The corresponding ROC AUC value for OSN and OSR is only 0.66 (with 95% confidence interval [0.53,0.79]), which is only partially better than the baseline score of ≈0.5 expected by chance, again indicating that the groups are very similar.

### 2.4. In What Ways Are the Groups Different?

Having found evidence that the N and L groups are different, and quite markedly so, we now study *in what ways* the groups are different, i.e., which cytokines are most responsible for the differences. Please note that we will not do the same for the OSN and OSR data, because we did not find a significant difference between those groups. The relative contribution of each variable (cytokine) in distinguishing groups is known as *variable importances*. Here we measure the variable importances in two different ways.

To prepare the data, we process the cytokine data so that each variable is log10 transformed, and centered and scaled so that each variable has mean 0 and standard deviation 1. It is important to do ensure all variables are on the same scale, to facilitate easy and meaningful comparison between variables. After this processing, we compare the centroid of the N samples to the centroid of the L samples, i.e., compare the mean values of each cytokine in the N group, to the mean values of the variables in the L group. Because the data have been preprocessed, comparing means is mathematically reasonable.

**Figure 2:**
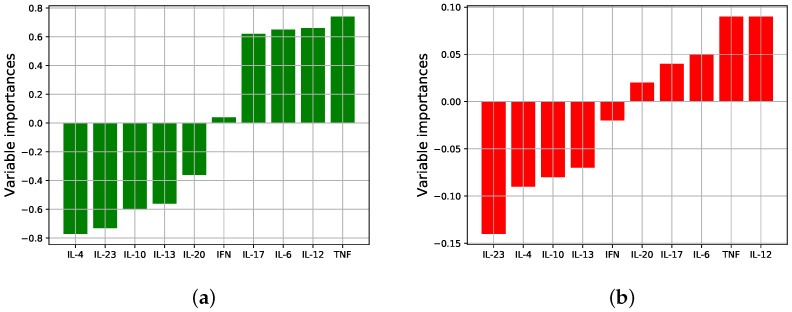
Variable importances (signed): (**a**) Comparing N and L group centroids (mean values of each variable); and (**b**) using regression coefficients from the PLSR model. The two methods largely agree in terms of which cytokines are most important and in whether up/down regulated.

Figure 2a shows the signed variable importances obtained from the centroids, where positive signs/values indicate up-regulation of the cytokine in the L group relative to N, and negative signs/values indicate down-regulation. As can be seen, several of the cytokines are up/down-regulated in the different groups, and the magnitude of the difference varies between cytokines, i.e., not all cytokines are equally important in distinguishing the groups. It appears from the figure that the most important (by largest magnitude) cytokines distinguishing the groups are IL-4 (down), TNF-*α* (up), IL-23 (down), and IL-12 (up) with the other cytokines of slightly lesser consequence.

The centroid method just examined affords easy-to-interpret importances, but it lacks any ability to account for correlations between cytokines, because each cytokine mean value is treated separately to each other mean value. Therefore, secondly, we measure variable importances via the coefficients of PLSR prediction model (Methods). These coefficients can account for correlations and linear combinations of cytokines which associate to each group. Figure 2b shows these (signed) importances, and it is apparent that there is close agreement between the two methods regarding which cytokines are most significant, and whether up/down regulated. Having said that, there is some difference, between Figure 2a,b, namely that in (b) more importance has been placed on IL-23 and IL-12 in contrast to (a), with the other cytokines considerably less important.

## 3. Discussion

The rationale for the measurement of cytokines in osteoporosis is that cytokines have been shown unequivocally to play major roles in numerous autoimmune diseases, hypersensitivity reactions and pathologic conditions, such as rheumatoid arthritis, insulin-dependent diabetes, multiple sclerosis, dermatitis and asthma to name but a few. This manuscript adds on tools that could be used to explore possible associations between profiles of cytokines with osteoporosis and osteopenia in postmenopausal women. If the tools and results are confirmed in larger sample sizes, we might be closer to be able to claim and conclude that (i) some cytokine profiles can indeed serve as surrogate markers for the risk of developing osteoporosis, (ii) follow up on progression of the disease/therapy and (iii) that these observations may lead to the development of new preventive therapies. The last point is validated by the fact that effective and safe treatment of several autoimmune diseases such as rheumatoid arthritis, inflammatory bowel diseases, multiple sclerosis etc. are now based on blocking inflammatory cytokines and/or their receptors [24,25].

Cytokines are known to work in a complex hierarchical network and most of them show pleiotropic, redundant and synergetic actions, making the full understanding of the balance challenging. While several osteoporosis reports have suggested the use of cytokines as potential biomarkers, a single biomarker may be insufficient; thus, it may be more appropriate to use a multivariate approach. We compared multivariate cytokine profiles of postmenopausal women with normal and low BMD, as well as comparing osteopenic to osteoporotic women, using several statistical techniques. These data have previously been studied in terms of individual cytokine levels and simple ratios, but we sought to investigate these data further. Studying all tested cytokines, our main aims were to see if the contrasting groups were different, and if so, how strongly different and which cytokines were most responsible for the differences.

The main results of our multivariate analysis were that none of the groups appear to have subgroups/clusters, and that normal- and low-BMD subjects have large differences—hence presumably clinically significant—as well as statistically significant (*p*-value = 0.00013) differences in cytokines values, with IL-23, IL-12, TNF-*α*, IL-4 and IL-6 showing pronounced differences between normal- and low-BMD cases. On comparing osteopenic to osteoporotic samples, no significant difference was found between cytokine values (*p*-value = 0.81). It is interesting that no significant difference was found, even though there was a difference found between N and L groups. The lack of evidence for a difference may possibly be due to the relatively small sample sizes of the OSN (*n* = 31) and OSR (*n* = 15).

Knowing which cytokines are most important in separating the normal- and low-BMD groups (i.e., the variable importances) may lead to future trials to administer cytokine blockers [26]. Indeed, cytokine treatment and blocking is used in many diseases [24,25], and may be applicable in the case of low-bone-density patients. Furthermore, managing osteoporosis by regulating the cytokines linked to symptoms via minimum modulators [27] may be more easily achievable, and more effective, due to focusing narrowly on just the few cytokines with greatest importances, as we have done here. Finally, another benefit of our work is to aid in the basic science of osteoporosis, including the pathologic sequence of bone loss. Relatedly, our identification of relevant cytokines suggests these as biomarkers for both indicating and quantifying a risk of developing osteoporosis, and for monitoring the efficacy of a treatment course.

While there is no doubt that menopause triggers rapid bone loss in women, what remains unclear is the proportion of bone loss attributable to direct effects of estrogen versus indirect effects attributable to other potential factors such as cytokines. In other words, can other factors such as proinflammatory cytokines act as significant mediators in estrogen-deficiency bone loss? A supportive observation comes from the work of Charatcharoenwitthaya and colleagues, who administered cytokine blockers to early postmenopausal women who ceased transdermal estrogen therapy, and found that blockade of IL-1 or TNF-*α* resulted in a reduction of estrogen-deficiency-induced bone resorption by 50% [26]. These experiments in humans are supported by several reports on animal knock-out models. IL-1 receptor-deficient or TNF-deficient mice showed no significant bone loss after ovariectomy as compared to the wild type [28,29] and ovariectomy-induced estrogen deficiency did not bring about any change in bone mass or rates of remodeling in IL-6-deficient mice [30].

In addition to changes of cytokine production by monocytes, T cell abnormalities have been reported in patients with osteoporosis. Fujita et al. [31] described an increased CD4+/CD8+ ratio in osteoporosis; these findings were corroborated by Imai et al. [32] and Rosen et al. [33]. Hustmyer et al. [34] described a negative correlation between the CD8+/CD56+ subset and bone mineral density. It was also reported that in postmenopausal women with osteoporotic fractures the CD8+/CD57+ subset is expanded; moreover, in fracture patients the percentage of CD8+ cells that expressed TNF-*α* was augmented [35]. Thus, in addition to monocytes and their products, T cells appear to contribute to the pathogenesis of primary osteoporosis.

We realize that this study has several limitations. From a physiological point of view, measuring the production of cytokines by PBMC in vitro only partly reflects the much more complex situation in vivo, where T lymphocytes are in close contact with other cells and factors including regulatory T lymphocytes, natural killer cells, cytokines and their receptors. We have tested only 10 cytokines, and cytokine measurements are subject to limitations related to patient, pre-analytical variability and analytical variability [36]. We recognize that the statistical power of analysis is dependent on sample numbers, and there is an increased risk of “under-power” or observing false negative results when analyzing high-dimensional data with just a few samples (i.e., many cytokines tested in a small number of samples). Given that our data set is based on only 71 women, and that the data has several variables (i.e., several cytokines), the findings and associations should be taken as tentative results, subject to future corroboration. Also, the data presented here does not prove causation. Having said that, our analysis and findings do point to several interesting avenues to be explored further. Finally, while standard practice, the fact that low-BMD women are routinely prescribed vitamin D and calcium supplements, but not normal BMD, may also introduce some limitations in our analysis.

It is worth mentioning that the full understanding of the pathogenesis of osteoporosis is incomplete. Several researchers suggest that bone loss may be due to several age-related factors which in addition to changes in the ovaries, adrenal gland and kidney may include other factors such as age-related oxidative stress, genetic predisposing factors as well as immune and inflammatory mediators [37,38]. Hence, examining these factors as well as obtaining and analyzing larger data sets to confirm/refute our findings are left for future work.

## 4. Materials and Methods

### 4.1. Patient Selection, Demographics, and Ethical Approval

The data used in this work were originally collected by the authors of Ref. [20]; full details of data collection methods can be found therein. Here we give a summary of the data collection process and demographics.

A group of 71 postmenopausal women were studied (i.e., absence of menstrual periods for at least 12 months prior to the study). All participants were recruited from the Physical Medicine Unit at Mubarak Al-Kabeer Hospital, Kuwait and underwent clinical assessment by a single recruiter. These were grouped as women with normal BMD (N, *n* = 25) or with low BMD (L, *n* = 46). Participants were further grouped by the T-scores of BMD into three groups: the normal group (N, T-scores > −1, *n* = 25), the osteopenia group (OSN, −2.5 < T-scores < −1, *n* = 31) and the osteoporosis group (OSR, T-scores < −2.5, *n* = 15). This was based on the guidelines set by the WHO and Adult Official Positions of the International Society for Clinical Densitometry (ISCD) (http://www.iscd.org/official-positions/2015-iscd-official-positions-adult/) as updated in 2015. All participants were interviewed, evaluated clinically and their demographic data such as age, weight, height, body mass index (BMI) and period since menopause were recorded on the day of examination. Women who were on systemic corticosteroids, malignancy, hyperparathyroidism, severe renal impairment, liver disease or experiencing any infectious disease were excluded due to possible effects on immune function and cytokine production. Women who needed calcium and vitamin D supplementation were receiving 600 mg of calcium and 200 IU of vitamin D twice daily.

This study was granted ethical approval from the Health Sciences Center of Kuwait University, and all participants gave written informed consent prior to participating in the study.

A summary of the demographics is given in the Supplementary Figure, which shows a table taken from Ref. [20]. Because of the possibility of confounding factors in the statistical comparison of groups, Azizieh et al. [20] used regression analysis to exclude age as a confounding factor and years since menopause. BMI were comparable between women with normal BMD and women with low BMD.

### 4.2. Data Preprocessing and Missing Values

Missing cytokine values were replaced with the median values of the sample, which is a conservative technique, not biased towards specific characteristics of the data. In total, 52 of the 71 women had at least one missing cytokine value, out of the 10 measured for each woman. In more detail, 89/(71 × 10) = 13% of the data values were missing, which is not an overly large fraction. Having said that, of course the larger the fraction of missing values, the harder it is to detect statistical trends or differences in groups.

All concentrations were log10 transformed, because a log scale is a more natural scale to study cytokine concentrations on. Log transforming is also standard in the literature. After this, the data were centered (by subtracting the mean) and scaled (divided by the standard deviation).

### 4.3. Statistical Analysis

A *p*-value of < 0.05 was considered statistically significant in this study. Statistical analysis was done with the Python machine learning package Scikit-learn [39] using the iPython interface [40], and also in R Studio [41].

### 4.4. Subgroup Analysis

The Gap Statistic [21], implemented on R Studio [41], was used to estimate the number of subgroups in multivariate data. The recommended criterion for determining the number *k* of clusters in a sample was used, namely the smallest *k* such that Gap(*k*) ≥ Gap(*k* + 1) − 1 standard deviation. If *k* = 1, then there are no subgroups. Searching for subgroups in relatively small multivariate data samples can be challenging, nonetheless, we can expect to find subgroups if there is clear clustering.

### 4.5. Cramer Test

Multivariate comparison of the cytokine distributions between each of the comparison groups was done using the Cramer Test [22] on RStudio [41]. 100,000 bootstrapped samples were used, which is ten times larger than the default number.

### 4.6. PLS-DA

Partial least squares discriminant analysis (PLS-DA) is used when partial least squares regression (PLSR) is applied to problems with categorical outputs. Here the outputs/responses are the groups (e.g., N and L), so we use a 1/0 binary output for *Y*. Specifically, we use ‘0’ for N and ‘1’ for L, and ‘0’ for OSN and ‘1’ for OSR. The linear model is then *Y* = *Xβ* + *ζ*, where *X* is the data matrix (i.e., a matrix whose rows correspond to each patient, and whose columns represent the different cytokines), *β* is a column vector of the model coefficients (which represent the contribution of each cytokine to distinguishing the groups), and *ζ* the error term vector (which accounts for errors in the binary classification predictions).

When used as a dimension reduction projection, or ‘shadow’ of the data, the PLS-DA method will plot a 2D projection of the multivariate data of all cytokines tested. The algorithm projects the data onto two axes (latent variables 1 and 2), which are chosen optimally by the algorithm to best separate the two groups. The model coefficients vector *β* can be used for variable importances. To see why, if one cytokine is up-regulated for the group labeled ‘1’, then the corresponding element of *β* will be positive, and negative if down-regulated. If a cytokine is neither up- nor down-regulated between groups, then the corresponding element of *β* will be roughly 0.

## Supplementary Materials

Supplementary File 1

## Abbreviations

The following abbreviations are used in this manuscript:

MDPI	Multidisciplinary Digital Publishing Institute
DOAJ	Directory of open access journals
TLA	Three letter acronym
LD	linear dichroism
