# Supplementary material for: Multivariate Comparison of Cytokine Profiles for Normal- and Low-Bone-Density Subjects
Source: Diagnostics (Basel). 2019 Sep 30;9(4):134. doi: 10.3390/diagnostics9040134 (PMC6963703; doi:10.3390/diagnostics9040134)
Supplement: Supplementary File 1 [file diagnostics-09-00134-s001.zip › diagnostics-577499-supplementary.pdf]

	PM with Normal BMD (N) (n=25)	PM with Low BMD (L) (n=46)	p (N vs. L)	PM with Osteopenia (OSN) (n=31)	p (N vs. OSN)	PM with Osteoporosis (OSR) (n=15)	p (N vs. OSR)	p (OSN vs. OSR)
Age (years)	56.1+5.6	59.6+7.8	**0.03**	58.7+7.9	0.16	61.3+7.3	**0.001**	0.14
Weight (Kg)	80.5+12.2	75.5+12.7	0.13	75.7+12.1	0.55	75.2+12.3	0.18	0.96
Height (m)	158.7+5.2	156.1+6.1	0.15	157.3+5.3	0.18	153.5+6.9	**0.02**	0.58
BMI (kg/m^2)	32.0+5.2	31.0+5.0	0.47	30.6+5.0	0.28	31.8+5.1	0.9	0.4
Years since menopause (yr)	7.6+5.5	9.0+7.2	0.53	7.4+6.4	0.75	12.1+7.7	**0.06**	**0.019**
T-score hip	0.2+0.8	-1.4+0.87	**0.0001**	-1.1+0.8	**0.0001**	-2.0+0.7	**0.0001**	**0.001**
Hip BMC (g/cm^2)	1.02+0.12	0.78+0.12	**0.0001**	0.81+0.13	**0.0001**	0.73+0.1	**0.0001**	0.074
T-score L1-L4	-0.11+0.65	-2+0.65	**0.0001**	-1.7+0.4	**0.0001**	-2.7+0.5	**0.0001**	**0.0001**
L1-L4 BMC (g/cm^2)	1.17+0.1	0.90+0.1	**0.0001**	0.93+0.09	**0.0001**	0.83+0.9	**0.0001**	**0.003**
